# Display color gamut as a spectral risk factor for myopia: a unifying hypothesis linking chromatic emmetropization, cone circuitry dysfunction, and the spectral deficiency of digital screens

**DOI:** 10.3389/fopht.2026.1901460

**Published:** 2026-08-07

**Authors:** Pierpaolo Emanuele Maimone

**Affiliations:** Ophthalmology and Vision Science, Studio Maimone, S. Teresa di Riva, Messina, Italy

**Keywords:** color gamut, cone dysfunction, digital displays, emmetropization, longitudinal chromatic aberration, myopia, neuropsin, OPN5

## Abstract

The global prevalence of myopia in children rose from 24% in 1990 to 36% in 2023, with projections exceeding 39% by 2050. While time spent outdoors is robustly protective against myopia, the specific spectral properties of light responsible for this protection remain insufficiently characterized. We propose that the spectral power distribution (SPD) of modern digital displays may represent a previously unrecognized environmental risk factor for myopia through the systematic elimination of two wavelength bands critical to emmetropization. All current display technologies emit no measurable light below approximately 420 nm, entirely excluding the violet band (360–400 nm) that activates OPN5/neuropsin (λmax ≈ 380 nm) in retinal ganglion cells. Additionally, the narrow-band RGB primary architecture of displays distorts the cyan-green region (490–560 nm), potentially perturbing the SWS/LWS opponent chromatic signal that the Gawne-Norton dual-detector model identifies as the primary longitudinal chromatic aberration (LCA)-based drive for emmetropization. These two mechanisms are consistent with the dose-response relationship between screen time and myopia risk documented in large-scale epidemiological studies. The hypothesis is grounded in five converging lines of evidence: (1) the OPN5/violet light pathway; (2) the LCA dual-detector emmetropization model; (3) genetic evidence from Blue Cone Monochromacy and Bornholm Eye Disease; (4) red-light therapy trials; and (5) subgroup analyses by display device type. We propose that display color gamut — and the spectral power distribution it determines — currently optimized exclusively for colorimetric fidelity, merits evaluation as a modifiable variable in the global myopia control agenda.

## 1 Introduction

### 1.1 The myopia epidemic and the outdoor paradox

Myopia has emerged as a global public health crisis. A comprehensive meta-analysis of 276 studies documented an increase in prevalence from 24.3% in 1990 to 35.8% in 2023 among children and adolescents, with projections reaching 39.8% by 2050 — representing over 740 million affected children worldwide (1). The clinical burden extends beyond uncorrected distance vision: high myopia (≥−6 D) substantially elevates lifetime risk of retinal detachment, myopic maculopathy, glaucoma, and preventable blindness (2). A dose-response meta-analysis of 45 studies comprising 335,524 individuals confirmed a sigmoidal relationship between digital screen time and myopia risk, with OR increasing from 1.05 at 1 hour/day to 1.97 at 4 hours/day (3).

Among protective factors, time spent outdoors occupies a uniquely robust position. Rose et al. demonstrated in a landmark cohort of 2,353 Sydney schoolchildren that outdoor exposure was inversely correlated with myopia prevalence independently of near work load (4), and subsequent randomized controlled trials and meta-analyses have consistently confirmed reduced myopia incidence with increased outdoor time (5, 6). Yet the specific properties of outdoor light responsible for this protection remain incompletely characterized. The most authoritative current review — the International Myopia Institute (IMI) white paper on light and myopia (2025), synthesizing evidence through June 2025 — explicitly states that “evidence remains insufficient regarding how specific light properties, including spectral composition, affect human myopia,” and identifies the characterization of the spectral environment of digital devices as an open research priority (7). This gap is the starting point of the present hypothesis.

### 1.2 Historical context: from Cohn to the digital screen

The association between indoor near work and myopia predates modern science. Hermann Cohn’s 1886 monograph *The Hygiene of the Eye in Schools* documented myopia prevalence exceeding 58% in German gymnasium upper grades and attributed this to near work under poor indoor illumination (8) — figures comparable to current East Asian school-leaver rates. A recent historical review explicitly paralleled Cohn’s nineteenth-century observations with the contemporary myopia epidemic, concluding that “the younger the children are subjected to near work and the less time spent outdoors, the higher the prevalence of myopia” (9). What Cohn could measure in terms of illuminance, he could not measure in terms of spectral composition. It is precisely this spectral dimension — manifest in its most concentrated form in the emission profile of modern digital displays — that the present paper proposes as a previously uncharacterized and mechanistically specific environmental risk factor.

### 1.3 Prior work: the M-cone circuitry hypothesis

In 2008, the present author proposed in *Medical Hypotheses* that dysfunctional medium-wavelength (M/L) cone signaling may contribute to axial elongation, drawing on the clinical observation that Blue Cone Monochromacy (BCM), Bornholm Eye Disease (BED), and incomplete achromatopsia — all conditions disrupting M/L cone function — are strongly associated with myopia, whereas complete achromatopsia shows a hyperopic distribution (10). That letter called for research extending “the analysis to the broader spectrum of light radiation taking into account UVR as well as green and perhaps red and blue wavelengths.” The present paper formalizes and extends that hypothesis, integrating seventeen years of subsequent mechanistic discoveries around a specific, measurable, and technologically modifiable parameter: the spectral power distribution determined by the color gamut design of digital displays.

## 2 The spectral architecture of digital displays

### 2.1 Color gamut, primary selection, and spectral power distribution

**Figure 1:**
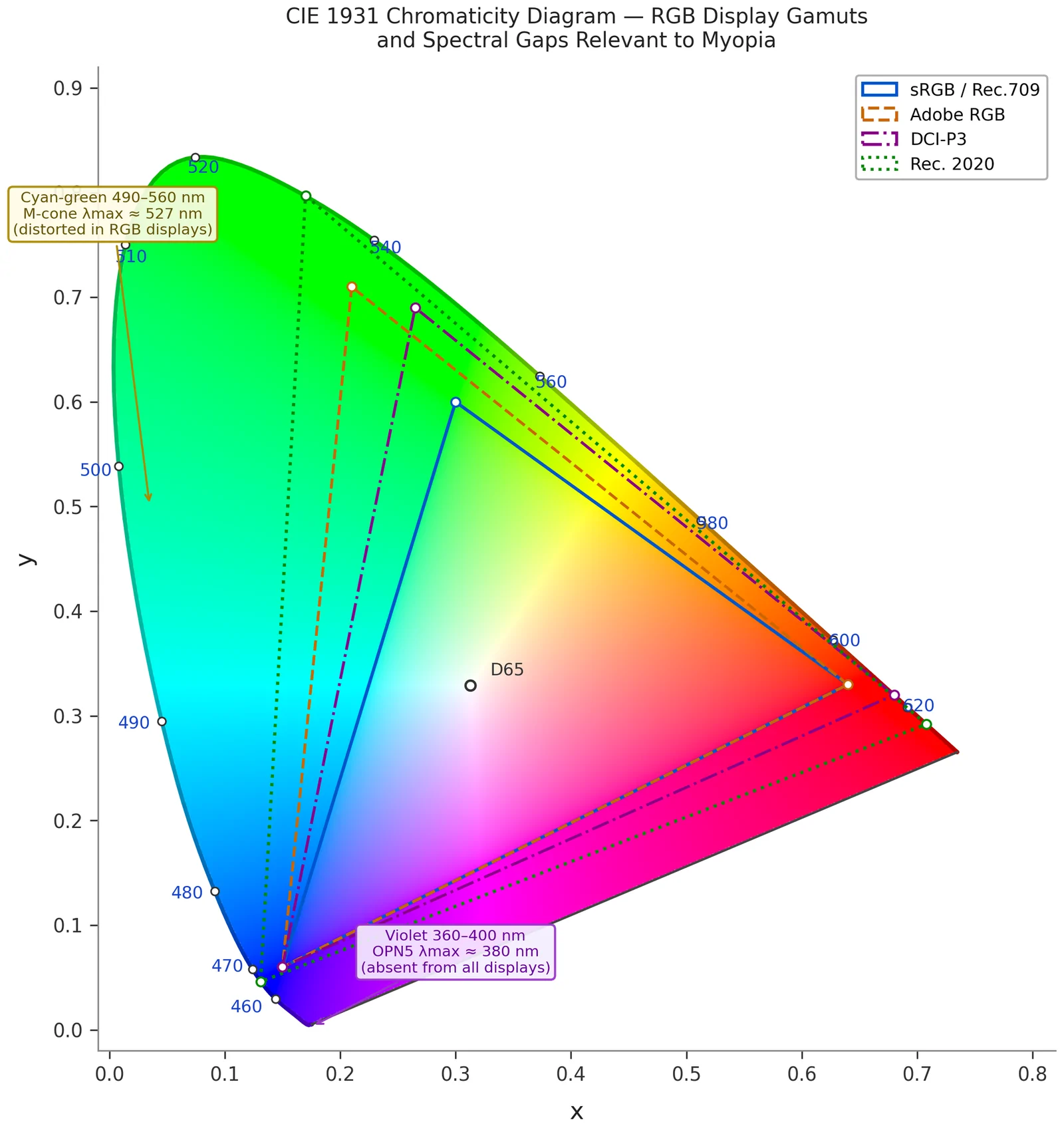
CIE 1931 xy chromaticity diagram showing the spectral locus (horseshoe), line of purples (lower boundary), and color gamut triangles for four major display standards. Note that geometric distance on the CIE 1931 chromaticity diagram represents differences in perceived color and does not directly encode photon flux, cone excitation ratio, or biologically effective retinal irradiance; the figure is used here to illustrate the primary architecture of display gamuts and the location of biologically relevant spectral regions, not to quantify the magnitude of biological spectral deprivation. The arguments concerning biological mechanisms in Sections 4 and 5 operate at the level of spectral power distribution and retinal photon flux. The cyan-green zone (490–560 nm, yellow annotation) is the area most geometrically distant from all RGB gamut triangles — the spectral region where M-cone sensitivity peaks (λmax ≈ 527 nm) and where the LCA-based chromatic emmetropization signal is most severely compressed by RGB primary architecture. The violet zone (360–400 nm, purple annotation) corresponds to the activation band of OPN5/neuropsin (λmax ≈ 380 nm) and is absent from the SPD of all current display technologies. D65: CIE standard daylight illuminant white point (x = 0.3127, y = 0.3290).

All modern digital displays produce color by additively mixing three narrow-band primary emitters: red, green, and blue (RGB). The *color gamut* of a display is the range of colors it can reproduce, defined as a triangle inscribed within the CIE 1931 xy chromaticity diagram (**Figure 1**). The three vertices of this triangle correspond to the chromaticity coordinates of the red, green, and blue primaries. Different display standards — sRGB (the universal consumer standard), DCI-P3 (cinema and Apple devices), and Rec. 2020 (HDR/UHD) — use different primary coordinates, creating triangles of different sizes.

A critical distinction must be drawn between the color gamut and the spectral power distribution (SPD) that it determines. The color gamut is a design parameter — the specification of which primary chromaticities a display will use — set at the engineering level. The SPD is the physical quantity that results from those primary choices: the distribution of radiant energy across wavelengths emitted by the display and reaching the viewer’s retina. The gamut determines the primaries; the primaries determine the SPD; and the SPD determines the biologically effective photon flux at each wavelength. Critically, displays with similar gamuts may have different SPDs depending on the underlying light-source technology (LED, OLED, quantum dot), and the biological mechanisms discussed in this paper — OPN5 activation, cone excitation balance, chromatic defocus signals — depend on the SPD at the retinal level, not on the colorimetric gamut per se. Throughout this manuscript, “gamut” is used when referring to the colorimetric design parameter or to display engineering contexts; “SPD” is used when the argument concerns photon flux, cone excitation, or retinal photobiological stimulation.

This trichromatic architecture is fundamental to all current display technologies regardless of panel type: LCD/LED backlights, OLED pixel arrays, and Quantum Dot (QD-OLED) displays all use three narrow-band primaries. Two aspects of the resulting SPD are directly relevant to myopia: the complete absence of violet radiation and the distortion of the cyan-green region.

### 2.2 Complete absence of violet radiation (360–400 nm)

All current display technologies emit no measurable light below approximately 420 nm. Their blue primaries peak at 440–460 nm — for LCD-LED displays, this corresponds to the InGaN blue LED peak; for OLED displays, to the organic blue emitter peak; for QD-OLED, to the blue LED that pumps the quantum dot layer. None approach the violet band (360–400 nm). This absence is compounded in indoor environments by UV-blocking coatings on building glass and corrective lenses, which filter the violet component of any residual ambient illumination.

Systematic spectroscopic measurements in Tokyo (2025) quantified outdoor violet irradiance at a mean of 310 μW/cm² annually, while indoor environments — even without displays — registered near-zero values due to UV-blocking glass and artificial lighting (11). Standard indoor LED and fluorescent lighting sources have their blue emission peaks at 445–460 nm, with negligible energy below 420 nm; incandescent sources are more red-shifted still. The display itself contributes zero violet photons. Screens compound this indoor violet deprivation: not only do they emit no violet photons, but prolonged screen use is associated with reduced outdoor time (3), further compounding the spectral deficit. During evening screen use — the period of greatest concern given the circadian gating of OPN5 function discussed in Section 4.1 — ambient illumination, building glass, corrective lenses, and the display itself converge to create a retinal violet photon environment approaching zero, across all contributing sources simultaneously. The net daily violet irradiance experienced by a heavily screen-exposed child in a modern indoor environment approaches zero.

### 2.3 Spectral distortion of the cyan-green region (490–560 nm)

The second spectral consequence of RGB primary architecture concerns the cyan-green region. As illustrated in **Figure 1**, the left side of the CIE 1931 horseshoe — corresponding to wavelengths 490–560 nm — is geometrically the most distant from any inscribed RGB triangle. This means that the chromaticities in this region are reproduced by displays as mixtures of blue and green primaries. The result is a spectral power distribution that is systematically LWS-dominant (long-wavelength biased) and SWS-deficient (short-wavelength biased) relative to broadband daylight. The M-cone, with its peak sensitivity at λmax ≈ 527 nm, falls within this compressed and distorted zone. As discussed in Section 4.2, this chromatic asymmetry has direct consequences for the LCA-based emmetropization mechanism.

### 2.4 Metameric equivalence does not imply chromatic defocus equivalence

A potential colorimetric objection to the display gamut hypothesis takes the following form: digital displays routinely achieve *metamery* — the production of an identical trichromatic cone excitation pattern to that of a natural broadband scene under daylight illumination. If a display can perceptually match a leaf, a face, or a blue sky, does the biological significance of spectral differences between the display and the original scene disappear? The answer is no, and the distinction is critical.

Metameric equivalence is defined at the level of integrated cone excitation: two stimuli are metamers if they produce identical (L, M, S) responses, regardless of their spectral composition. Trichromatic metamery does not imply equivalence at the level of wavelength-resolved retinal contrast — the signal on which the Gawne-Norton dual-detector model depends. The model operates on the *spatial image contrast* captured by SWS and LWS cone arrays as a function of wavelength-dependent focal plane depth. Because longitudinal chromatic aberration places each wavelength at a slightly different axial focus, the retinal image of a given spatial frequency contains different amounts of contrast in different spectral channels depending on the axial position of the retina relative to each focal plane. This wavelength-resolved contrast distribution is the emmetropization signal.

A display SPD composed of three narrow-band primaries (e.g., blue at 450 nm, green at 530 nm, red at 615 nm) and a broadband daylight SPD that are metameric under CIE photopic conditions will produce identical integrated (L, M, S) cone excitations. However, their wavelength-resolved contrast profiles at the retinal level are fundamentally different. The daylight SPD distributes energy smoothly across the full chromatic defocus gradient from 400 to 700 nm, producing a continuous wavelength-weighted contrast distribution across all focal planes. The display SPD concentrates its energy at three discrete wavelengths, each contributing contrast selectively to the focal plane corresponding to its peak wavelength. The resulting chromatic defocus signal — the ratio of SWS to LWS contrast as a function of retinal position — is therefore different between a display and daylight even when their trichromatic matches are identical. Metameric equivalence at the level of cone excitation totals does not protect against this SPD-level distortion of the emmetropization signal.

A secondary observation supports this argument: the gap between the spectral locus and the nearest RGB gamut boundary in the cyan-green zone (490–540 nm) is approximately 0.08–0.15 CIE xy units, representing 5–15 times the semi-major axis of a MacAdam just-noticeable-difference ellipse in that region (~0.010–0.015 xy units). The missing spectral regions are therefore additionally not perceptually recoverable by metamery — they lie outside the gamut and outside the perceptual reach of any metameric substitution. However, this geometric argument is secondary to the metabolic argument: even if metamery were achievable, the distortion of the wavelength-resolved chromatic defocus signal would persist.

## 3 The hypothesis

We propose that the spectral power distribution of modern digital displays — determined by their color gamut primary architecture — may represent a previously uncharacterized environmental risk factor for myopia by depriving the retina of two spectral inputs proposed to be critical for emmetropization regulation:

Deprivation Pathway I: The complete absence of violet radiation (360–400 nm) in display SPDs reduces retinal violet photon flux to effectively zero, removing the primary photonic input for OPN5/neuropsin activation (λmax ≈ 380 nm) in retinal ganglion cells, and thereby eliminating the OPN5-mediated choroidal thickening mechanism and EGR1-dependent suppression of axial elongation.

Deprivation Pathway II: The RGB primary architecture delivers a systematically LWS-dominant, SWS-deficient retinal SPD that perturbs the SWS/LWS opponent chromatic signal which, via longitudinal chromatic aberration (LCA), provides the principal spectral drive for emmetropization. This perturbation operates independently of trichromatic metamery, as discussed in Section 2.4.

These two pathways are proposed to operate simultaneously and to be amplified by the epidemiological relationship between screen time and myopia risk (3). Together, they generate the prediction that the display spectral power distribution — a parameter currently optimized exclusively for colorimetric fidelity — is a measurable and potentially modifiable contributing variable within the multifactorial etiology of childhood myopia. The spectral deficiencies, their biological targets, and their predicted emmetropization consequences are summarized in **Table 1**.

**Table 1:** Display SPD deficiencies relative to broadband daylight, biological targets, and predicted emmetropization consequences.

Spectral region	Status in display SPD	Biological target	Emmetropization consequence
360–400 nm (violet)	Completely absent (all technologies)	OPN5 (λmax 380 nm), EGR1, choroidal OPN5	Zero retinal violet photon flux; no OPN5-mediated choroidal thickening; loss of EGR1 axial brake
420–460 nm (blue)	Narrow-band peak only (blue primary)	SWS cone (λmax 420 nm)	Partial LCA signal; potential SWS/LWS imbalance depending on scene chromaticity
490–560 nm (cyan-green)	Distorted by RGB primary gap (see Section 2.4)	MWS cone (λmax 527 nm); SWS/LWS chromatic balance	Perturbed chromatic defocus gradient; SPD-level imbalance independent of trichromatic metamery
630–700 nm (far-red)	Present in display red primary, but at ~0.05–0.15 mW/cm² total — ~100× below clinical RLRL dose	Photobiomodulation pathway (cytochrome c oxidase, peak ~650 nm)	Insufficient irradiance for RLRL-equivalent choroidal stimulus; deficit is of dose, not spectral presence

## 4 Mechanistic support for the hypothesis

### 4.1 The OPN5/violet light pathway

OPN5 (neuropsin) is a non-visual opsin expressed in a subset of retinal ganglion cells (RGCs), with a peak spectral sensitivity of λmax ≈ 380 nm in humans — precisely within the violet band absent from all display SPDs (7). The relevant biological quantity for OPN5 activation is retinal photon flux at 360–400 nm: the number of photons delivered per unit retinal area per unit time in this band. For current displays this quantity is zero, regardless of total luminous output. The evidence for OPN5’s role in myopia control proceeds through a well-characterized molecular cascade:

Step 1 — Photoreception. Violet light activates OPN5-expressing RGCs. This activation is subject to circadian gating: evening violet light exposure is sufficient to produce full protection, suggesting that the circadian window of maximum OPN5 sensitivity coincides precisely with the period of peak screen use in school-age children (12).

Step 2 — EGR1 upregulation. OPN5 activation upregulates *EGR1* (Early Growth Response 1) in chorioretinal tissues. EGR1 is a zinc finger transcription factor that acts as a molecular brake on axial elongation (13).

Step 3 — Dopaminergic regulation. OPN5-expressing RGCs modulate retinal dopamine reuptake transporter activity, influencing vitreal dopamine levels. Dopamine is the primary anti-myopia neuromodulator in the retina (14).

Step 4 — Choroidal thickening (indirect). OPN5 activation via retinal RGC signaling promotes choroidal thickening (15).

Step 5 — Direct choroidal photosensitivity. A 2024 study in *PNAS* identified Ca²^+^ signaling responsive to violet light directly in choroidal capillary endothelial cells, mediated by OPN5 and other opsins expressed in the choroid itself (16). This second, independent choroidal route means violet light can modulate choroidal thickness without requiring retinal transduction.

Step 6 — Circadian clock regulation. OPN5 in RGCs regulates the phase of the autonomous retinal circadian clock, controlling photoreceptor disc shedding, gap junction coupling, and neurotransmitter release rhythms (7).

The cumulative retinal violet photon exposure during typical evening screen use approaches zero across all contributing sources. Indoor LED and fluorescent lighting has its blue emission peak at 445–460 nm with negligible energy below 420 nm; standard window glass and corrective spectacle lenses absorb strongly below 400 nm; and the display contributes no violet photons by design. The circadian gating of OPN5 sensitivity means that this deprivation occurs precisely when it matters most biologically. The combination of display SPD, indoor lighting, and window filtration creates a period of complete retinal violet deprivation that coincides with the window of peak OPN5 sensitivity.

Clinical evidence directly supports this cascade: children wearing violet-light-transmitting contact lenses showed significantly less axial elongation than those wearing standard violet-blocking spectacle lenses (13), and outdoor violet irradiance correlates inversely with myopia prevalence (11).

### 4.2 The LCA dual-detector emmetropization model

Postnatal emmetropization requires visual feedback signals encoding the *sign* of defocus (whether the retinal image is in front of or behind the photoreceptor layer). Longitudinal chromatic aberration (LCA) provides such a signal: the human eye refracts shorter wavelengths more strongly than longer ones, creating a differential focal plane shift of approximately 2.5 diopters across the visible spectrum (400–700 nm) (20). Short-wavelength light focuses closer to the lens; long-wavelength light focuses further back.

Gawne and Norton (2020) formalized this mechanism in the “opponent dual-detector spectral drive model” of emmetropization (17). The developing eye compares spatial image contrast captured by short-wavelength sensitive (SWS, λmax ≈ 420 nm) and long-wavelength sensitive (LWS, λmax ≈ 557 nm) cone arrays. Because LCA places their focal planes at different depths, the ratio of SWS to LWS image contrast encodes the sign of defocus. The emmetropization system drives axial growth toward the point at which SWS and LWS contrasts are balanced.

As discussed in Section 2.4, the narrow-band SPD of RGB displays distorts this signal relative to broadband daylight even when trichromatic metamery is achieved. A key experimental prediction of the model — confirmed in multiple animal species — is that spectrally asymmetric illumination disrupts the balance signal: tree shrews reared under narrow-band cyan light (505 nm) develop progressive myopia (18); narrow-band blue light similarly fails to support normal emmetropization (19). Both conditions remove one component of the SWS/LWS opponent pair from the continuous daylight chromatic defocus gradient. Human accommodative responses are also wavelength-dependent in ways consistent with LCA-based chromatic guidance (20).

Quantitative cone-excitation modeling using published display SPDs and Stockman-Sharpe 2-degree cone fundamentals (33) — proposed as an explicit first step in Section 8 — would test whether typical display chromaticities produce meaningful shifts in the SWS/LWS activation ratio relative to CIE D65 daylight. The current mechanistic argument is based on CIE chromaticity geometry and the qualitative reasoning presented above; such modeling would provide the quantitative confirmation needed to translate the hypothesis into a testable dose-response prediction.

### 4.3 Red-light therapy as confirmatory spectral evidence

Multiple randomized controlled trials demonstrated that twice-daily retinal irradiation with near-monochromatic 650 nm red light (1,600 lux, 3 minutes per session) significantly reduces axial elongation and induces choroidal thickening in myopic children (21–23), confirmed by meta-analysis (24). The proposed mechanism involves photobiomodulation of mitochondrial cytochrome c oxidase at its secondary absorption band in the red range.

This evidence is significant for the display gamut hypothesis in two respects. First, it establishes a clinical proof-of-concept that wavelength-specific retinal stimulation can control axial elongation through the choroidal axis. Second, it is important to note that standard displays *do* emit energy in the far-red range: display red primaries peak at approximately 615–635 nm (sRGB), 620–630 nm (DCI-P3), and 630–640 nm (Rec. 2020), with emission tails extending to 660–680 nm. However, the corneal irradiance delivered at typical viewing distances (approximately 0.05–0.15 mW/cm² total across the visible spectrum, of which perhaps 20–30% falls in the red primary band) is approximately two orders of magnitude below the ~5–8 mW/cm² employed in published RLRL clinical trials. Standard displays therefore lack not far-red energy per se, but the high absolute irradiance and narrow spectral concentration at 650 nm required to activate the photobiomodulation pathway at therapeutically relevant doses.

## 5 Genetic evidence from cone circuitry disorders

The hypothesis that M/L cone spectral signaling specifically regulates emmetropization — first proposed on clinical grounds in 2008 (10) — has since been supported by genetic evidence. This evidence provides the strongest available argument that chromatic spectral balance, not merely visual acuity or image quality, actively participates in axial growth regulation.

### 5.1 Blue cone monochromacy

BCM is an X-linked condition caused by mutations in the *OPN1LW/OPN1MW* gene cluster (chromosome Xq28), resulting in absent or non-functional L- and M-cone photopigments. BCM is characterized by high myopia as a nearly universal phenotypic feature. In a systematic analysis of axial length distributions across 19 genetically confirmed inherited retinal diseases, BCM had the highest odds ratio for increased axial length (25). Critically, this myopia develops in the absence of any optical defocus, demonstrating that the M/L cone spectral signal per se, independent of blur-driven emmetropization, is required to prevent axial elongation.

### 5.2 Bornholm eye disease

BED is caused by specific point mutations in the *OPN1LW/OPN1MW* opsin gene loci producing spectral shifts and reduced photopigment optical density of L/M cones. High myopia (MYP1 locus) is the defining clinical feature of BED (26). The myopia in BED arises from altered — not absent — cone spectral tuning, demonstrating that even subtle changes in the wavelength sensitivity of M/L cone photopigments are sufficient to disrupt emmetropization.

### 5.3 The critical dissociation: complete achromatopsia

Complete achromatopsia — in which all three cone photoreceptor types are absent and vision is entirely rod-mediated — shows a hyperopic rather than myopic refractive distribution (27). This dissociation is the key genetic argument: selective loss of M/L cone signaling (BCM, BED) produces myopia, while loss of all cone signaling (complete achromatopsia) produces hyperopia. This demonstrates that it is the specific *imbalance* created when the LWS/MWS component is lost while SWS signaling continues that drives myopia — not the absence of cone photoreception per se.

It is important to note that BCM and BED involve profound genetic and photoreceptor-level alterations in cone function — genetic ablation or structural alteration of photopigment synthesis from birth — whereas display SPD represents an environmental modulation of chromatic input to a genetically intact visual system. These are mechanistically distinct situations. The genetic evidence is cited here as a conceptual parallel at the level of chromatic signal imbalance: it demonstrates that the long-wavelength cone spectral channel plays a specific and necessary role in emmetropization, independent of optical blur, and that disruption of SWS/LWS chromatic balance — however caused — can produce myopia. The claim is not that display SPD imposes an equivalent condition to BCM, but that both create a chronic imbalance in the chromatic input that the emmetropization system uses to regulate axial growth.

## 6 Epidemiological evidence by display device type

If the display gamut hypothesis is correct, devices with greater SPD-level spectral distortion should confer higher myopia risk at equivalent screen time. Subgroup analyses by device type in epidemiological studies provide preliminary, indirect evidence consistent with this prediction.

A meta-analysis of 19 studies (n = 102,360) found that computer use showed the strongest association with myopia (categorical OR = 8.19, 95% CI: 4.78–14.04; per-hour OR = 1.22), television use was significant but weaker (categorical OR = 1.46), and smartphone use alone did not reach significance in cross-sectional studies (28). A second meta-analysis found that combined smart device use yielded OR = 1.26, rising to OR = 1.77 when combined with computer use (29). A longitudinal COVID-19 study found smartphone use associated with OR = 2.02 and computer use with OR = 1.81 (30).

These differentials are not fully explained by viewing distance alone. The display gamut hypothesis offers a complementary interpretation. LCD-LED monitor and television backlights (W-LED) have their blue pump at approximately 440–455 nm — 15–20 nm closer to the S-cone peak (λmax ≈ 420 nm) than OLED smartphone blue primaries (~455–465 nm) (34, 35). This means LCD-based devices stimulate S-cones more strongly per unit of perceived brightness, potentially creating a greater SWS/LWS imbalance. No existing study has measured and stratified by device SPD rather than device category — a methodological gap that directly limits interpretation.

## 7 Implications

### 7.1 Generalizability

The hypothesis applies to all children and adolescents using RGB-primary displays — currently the universal standard across all consumer electronics categories. The spectral deficiencies described are device-class properties of the SPD, shared by all sRGB, DCI-P3, and Rec. 2020 displays regardless of manufacturer, price point, or panel technology. The biological targets (OPN5 at 380 nm, SWS/LWS balance) are conserved across human populations. The hypothesis makes no claim to be the dominant driver of the myopia epidemic — near-work distance, accommodation demand, peripheral defocus, and outdoor time reduction are all established contributing factors. The present hypothesis proposes a previously uncharacterized additional spectral dimension within this multifactorial framework.

### 7.2 Technological modifiability

Uniquely among environmental myopia risk factors, the display SPD is a design parameter. Primary selection — the choice of which wavelengths to use for the red, green, and blue emitters — is made by manufacturers and is in principle adjustable. Violet-supplemented backlight architectures, adding a fourth violet LED component (360–400 nm) to existing RGB configurations, are technically feasible without altering perceived white balance. A four-primary micro-LED display proposal (2025) explicitly optimized for photobiological health criteria including OPN5 activation demonstrates that the display engineering community is receptive to photobiological considerations in primary selection (31).

The practical feasibility of violet supplementation at display luminance levels requires quantitative grounding. Animal model evidence for OPN5-mediated myopia suppression (Jiang et al., 2021) was obtained at corneal violet irradiances of approximately 10–100 μW/cm² at 380 nm. Outdoor violet irradiance has been measured at a mean of 310 μW/cm² annually (Kondo et al., 2025) (11). A violet-supplemented display adding a narrow 380–400 nm LED component at approximately 1% of total luminous output would deliver approximately 3–15 μW/cm² of violet corneal irradiance at typical viewing distances — within the range of effective doses in animal models. This level is well below the ICNIRP and ANSI Z136 photochemical hazard limits for near-UV exposure (approximately 1–10 mW/cm² for occupational extended exposure), providing a photo safety margin of two to three orders of magnitude. These estimates must be qualified: direct human retinal OPN5 activation thresholds have not been measured *in vivo*, and cross-species extrapolation from mouse models carries the usual uncertainties. The ongoing NCT06501131 clinical trial will provide relevant *in-vivo* evidence on retinal responses to controlled spectral manipulation. If the gamut hypothesis were validated, it would create a rationale for “myopia-informed” display specifications — an intervention deployable at the point of manufacture, without requiring behavioral change.

### 7.3 Spectral reporting in myopia research

The IMI 2025 review calls for reporting “radiant power emitted at each wavelength” in light and myopia studies (7). We extend this recommendation to include SPD characterization of display devices as a standard covariate in all pediatric myopia studies assessing screen time. The current practice of categorizing screen time by device type without measuring SPD conflates devices with potentially very different photobiological impacts.

## 8 Hypothesis testing

### 8.1 Cone-excitation modeling (computational first step)

Prior to prospective clinical testing, a computational analysis using published display SPDs and Stockman-Sharpe 2-degree cone fundamentals (33) should quantify the SWS/LWS excitation ratio for representative device categories (LCD-LED television, OLED smartphone, wide-gamut monitor) relative to CIE D65 daylight. Such modeling would establish whether typical display chromaticities produce meaningful shifts in the SWS/LWS activation balance in the direction proposed, providing the quantitative foundation for clinical dose-response predictions. This analysis is technically feasible with existing published SPD data (34, 35) and published cone fundamentals, and is proposed as an immediate first step.

### 8.2 SPD characterization of consumer displays by category

Systematic spectrophotometric measurement of representative devices should quantify violet photon flux (360–400 nm) and the SWS/LWS activation ratio as a function of device type and gamut standard. An active clinical trial (NCT06501131) testing partial spectral absence in LED lighting on ocular parameters (32) provides a directly transferable methodology.

### 8.3 Retrospective epidemiological analysis with SPD stratification

Existing pediatric myopia cohort data should be re-analyzed stratifying by measured device SPD rather than device category. The prediction is that SWS/LWS activation ratio and violet photon flux will show stronger associations with myopia progression than device category alone, after adjustment for viewing distance and total screen time.

### 8.4 Prospective clinical trial of violet-supplemented displays

A randomized trial comparing standard displays vs. displays supplemented with a narrow violet LED component (360–400 nm) in children over 2 years, with axial length and sub foveal choroidal thickness as primary endpoints. The success of violet-light-transmitting contact lenses in reducing axial elongation (13) provides the mechanistic proof of concept.

### 8.5 Standardized spectral reporting

All future pediatric myopia studies assessing screen exposure should report device SPD alongside illuminance, screen time, and viewing distance, enabling the field to move from correlational to mechanistic analysis of display-related myopia risk.

## 9 Limitations

The mechanistic evidence linking display SPD to myopia is currently inferential, and the epistemic status of the proposed mechanisms must be stated clearly. No study has directly measured OPN5 activation as a function of display SPD, or correlated display spectral composition with axial growth biomarkers in human subjects. The cone-excitation modeling proposed in Section 8 — the necessary quantitative step connecting display SPD to the SWS/LWS imbalance argument — has not yet been performed for consumer devices specifically, and the current cyan-green argument rests on CIE chromaticity geometry and qualitative reasoning rather than calculated cone excitation ratios. These are significant gaps that define the immediate empirical agenda.

The IMI 2025 review cautions that species-specific differences in ocular transmittance and opsin tuning complicate the translation of animal findings to humans (7). Human lens transmittance below 400 nm decreases substantially with age, attenuating OPN5-relevant violet flux reaching the retina in adults; this factor is least consequential during the pediatric period targeted by the hypothesis but merits consideration. The human retinal OPN5 activation threshold has not been measured directly *in vivo*; the irradiance estimates in Section 7.2 are extrapolated from mouse models and carry the usual cross-species uncertainties.

The differential myopia risk by device type observed in epidemiological studies may reflect confounders — session duration, viewing distance, content type, ambient illumination — rather than spectral differences; current evidence is insufficient to isolate SPD effects. The RLRL therapy evidence operates at irradiances not comparable to ambient display emission, limiting quantitative extrapolation. The Gawne-Norton dual-detector model was developed and validated primarily in tree shrews and guinea pigs; while its predictions are consistent with human accommodative data (20), direct validation of the full emmetropization prediction in humans remains incomplete. These limitations do not invalidate the hypothesis but define the empirical work required to test it rigorously.

## 10 Conclusion

From Cohn’s nineteenth-century schoolrooms to twenty-first-century screen-saturated childhoods, myopia has consistently tracked with indoor near work under spectrally impoverished illumination. The display color gamut — and the spectral power distribution it determines — represents the most precisely characterizable and technologically modifiable instance of this spectral impoverishment, reducing violet radiation (the OPN5 activation band) to effectively zero and potentially distorting the SWS/LWS chromatic balance through the fundamental constraints of RGB primary architecture. Critically, these effects operate at the level of the physical SPD reaching the retina, independently of whether the display achieves perceptual metamery for natural scenes.

Building on a hypothesis first proposed in 2008 regarding the role of M/L cone circuitry in axial growth regulation (10), and integrating converging evidence from OPN5 photobiology, LCA-based emmetropization models, cone dysfunction genetics, and red-light therapy trials, we propose that display spectral power distribution is a measurable, biologically plausible, and potentially addressable variable in the global myopia control agenda. The investigation of this hypothesis requires spectrophotometry, cone-excitation modeling, and ocular biometry applied to an exposure that billions of children currently experience daily — and the design intent to ask, for the first time, whether the spectral composition of the light emitted by a child’s screen influences the trajectory of their eye’s growth.

## Data Availability

All original contributions are fully contained within the published article and its supplementary materials. No external data repository is required. The Python code used to generate **Figure 1** (CIE 1931 chromaticity diagram) is available upon reasonable request to the corresponding author at gebre@tiscali.it.
